# Effect of Fiber Esterification on Fundamental Properties of Oil Palm Empty Fruit Bunch Fiber/Poly(butylene adipate-*co*-terephthalate) Biocomposites

**DOI:** 10.3390/ijms13021327

**Published:** 2012-01-27

**Authors:** Samira Siyamak, Nor Azowa Ibrahim, Sanaz Abdolmohammadi, Wan Md Zin Wan Yunus, Mohamad Zaki AB Rahman

**Affiliations:** 1 Department of Chemistry, Faculty of Science, University Putra Malaysia, UPM Serdang, Selangor 43400, Malaysia; E-Mails: s.abdolmohammadi@yahoo.com (S.A.); mzaki53@gmail.com (M.Z.A.R.); 2 Chemistry Department, Center for Defence Foundation Studies, National Defence University of Malaysia; E-Mail: wanmdzin@upnm.edu.my

**Keywords:** oil palm EFB fiber, poly(butylene adipate-*co*-terephthalate), biocomposite, fiber esterification, thermal and mechanical properties, succinic anhydride

## Abstract

A new class of biocomposites based on oil palm empty fruit bunch fiber and poly(butylene adipate-*co*-terephthalate) (PBAT), which is a biodegradable aliphatic aromatic *co*-polyester, were prepared using melt blending technique. The composites were prepared at various fiber contents of 10, 20, 30, 40 and 50 wt% and characterized. Chemical treatment of oil palm empty fruit bunch (EFB) fiber was successfully done by grafting succinic anhydride (SAH) onto the EFB fiber surface, and the modified fibers were obtained in two levels of grafting (low and high weight percentage gain, WPG) after 5 and 6 h of grafting. The FTIR characterization showed evidence of successful fiber esterification. The results showed that 40 wt% of fiber loading improved the tensile properties of the biocomposite. The effects of EFB fiber chemical treatments and various organic initiators content on mechanical and thermal properties and water absorption of PBAT/EFB 60/40 wt% biocomposites were also examined. The SAH-g-EFB fiber at low WPG in presence of 1 wt% of dicumyl peroxide (DCP) initiator was found to significantly enhance the tensile and flexural properties as well as water resistance of biocomposite (up to 24%) compared with those of untreated fiber reinforced composites. The thermal behavior of the composites was evaluated from thermogravimetric analysis (TGA)/differential thermogravimetric (DTG) thermograms. It was observed that, the chemical treatment has marginally improved the biocomposites’ thermal stability in presence of 1 wt% of dicumyl peroxide at the low WPG level of grafting. The improved fiber-matrix surface enhancement in the chemically treated biocomposite was confirmed by SEM analysis of the tensile fractured specimens.

## 1. Introduction

The perspective of environmental concerns and lack of biodegradability all across the world, have initiated a drive for biodegradable materials, especially biodegradable polymers, as an alternative for holistically sustainable economic growth [1]. Among the commercially available biodegradable polymers, synthetic aliphatic and aromatic biopolyesters such as poly(butylene succinate-*co*-adipate) Bionolle, poly(3-hydroxybutyrate-*co*-3-hydroxyvalerate) Biopol^®^, the co-polyesters based on 1,4-butanediol adipic acid and terephthalic acid, Eastar-Bio^®^ and poly(butylene adipate-*co*-terephthalate) Ecoflex^®^, have shown similar physical and mechanical properties of the non-biopolymers [2].

Nowadays, the biodegradable polymer researches focus on cost reduction of biodegradable plastics. In this regard, mixing low-cost natural fillers with the biodegradable polymer has become an interesting alternative solution, and in recent times, plants fibers, including lignocellulosic fillers, have been receiving considerable attention [2,3]. Besides the high cost, the other major challenges of using biodegradable polymers in industrial application are the limitations of mechanical properties, and the life range application [4]. Lignocellulosic fibers have unique features such as low density, high mechanical properties like high toughness, enhanced energy recovery and biodegradability [3–6]. Furthermore, being inexpensive and having load bearing potential, natural fiber based composites have been extensively used in various sectors, including construction, aircraft and packaging [3,5]. Nevertheless, the natural fibers disadvantages, such as the poor moisture resistance and the propensity to form aggregates at some points in the processing trend [7], restrict the use of natural fibers as reinforcement in biodegradable polymers [8]. The biofibers hydrophilic nature is their major drawback for being used in biocomposites, which reduces natural fibers compatibility with hydrophobic polymer matrix throughout composite fabrication [6]. The incompatibility of components in composite systems causes poor composite performance and necessitates the use of chemical treatments in order to enhance the matrix and fiber adhesion [5]. Introducing chemicals possibly will activate hydroxyl (−OH) groups on fiber and/or present new moieties which can efficiently improve interlocking within the plastic matrix. Proper chemical treatment can improve the adhesion at the matrix/fiber interface and reduce the water absorption ability of a composite. These will inevitably enhance the fundamental properties of natural fibers reinforced biocomposites [7,8]. The practicability of using oil palm fibers as reinforcement in biopolymer composites as well as their effects on the mechanical properties of biodegradable thermoplastic polymer matrices has been extensively investigated in recent years.

Oil palm empty fruit bunch (EFB) fiber, mesocarp fiber and oil palm frond are some of the biomass wastes that have been extensively used in recent research works. EFB fiber possesses better mechanical properties and lower water absorption compared to mesocarp and oil palm frond [7,9]. Researchers have also studied the effects of chemical treatment of EFB fiber using various anhydrides, such as acetic, propionic and succinic anhydride, on mechanical and physical properties, like water absorption of EFB fiber reinforced polyester biocomposites [10]. They reported that fiber surface chemical treatments enhanced the mechanical properties and reduced the water absorption of polyester biocomposites compared to the unmodified EFB fiber reinforced polyester composite. The fibrous wastes were applied as reinforcement in biopolymer composites to develop biodegradable composites, in response to the existing demand for low cost stiff materials [7].

A systematic study was carried out to understand the behavior of EFB fibers before and after chemical treatment in poly(butylene adipate-*co*-terephthalate) (PBAT) based biocomposites. The present study focuses on treating EFB fibers with succinic anhydride (SAH) and incorporates esterified EFB fiber in PBAT to generate a new biocomposite with high thermal and mechanical performances.

## 2. Results and Discussion

### 2.1. FTIR Spectroscopy

**Figure 1:**
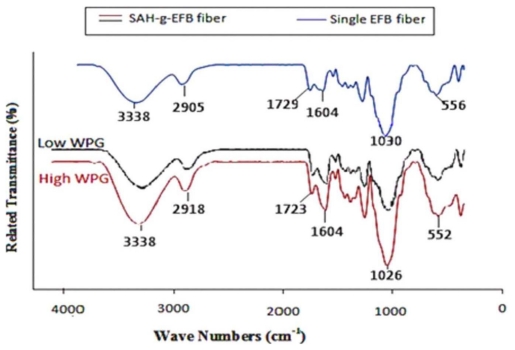
Comparison of FT-IR spectra of esterified oil palm empty fruit bunch (EFB) fiber at 120 °C in high and low weight percentage gain (WPG) and untreated EFB fiber.

The FTIR spectra (Figure 1) depict the changes of functional groups of EFB fiber before and after succinic anhydride modification. There is a decrease of absorption in the region of 1705–1750 cm^−1^ corresponding to the C=O stretching of carbonyl and ester groups. The peak within this region is attributed to the occurrence of ester bond within the carboxylic group of the fiber. The weakening of this peak corresponds to the extent of esterification; lower value in weight percentage gain (WPG) depicts lower peaks. Similar trend of esterification reaction was also observed around 1020–1040 cm^−1^ region attributed to the C=O stretching vibration of aliphatic primary and secondary alcohols in cellulose, hemicellulose, lignin and extractives, aromatic primary and secondary alcohols in lignin and extractives. Peaks for both of these regions proved very sensitive to any changes of reaction time and the resultant WPG. Higher WPG value was depicted with a higher band pattern. The decreasing rate of reaction at the 3200–3600 cm^−1^ region is attributed to the reduction of OH groups through substitution reaction as rejected by the increment in WPG value [11].

**Scheme 1:**
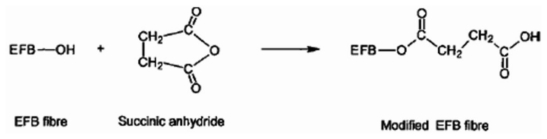
The possible reaction of EFB fiber with succinic anhydride.

The SAH treated samples exhibit a narrower peak at 3200–3600 cm^−1^, corresponding to O–H stretching vibration, as compared to the untreated one. The differences show that due to the reaction between SAH and the fiber lignocellulosic OH groups have been freed from the hydrogen bonding. The unreacted hydroxyl groups may cause these differences in the absorption at 3200–3600 cm^−1^ [12]. Scheme 1 illustrates the proposed chemical reaction for modification of EFB fiber with succinic anhydride, confirmed with the IR spectra.

**Figure 2:**
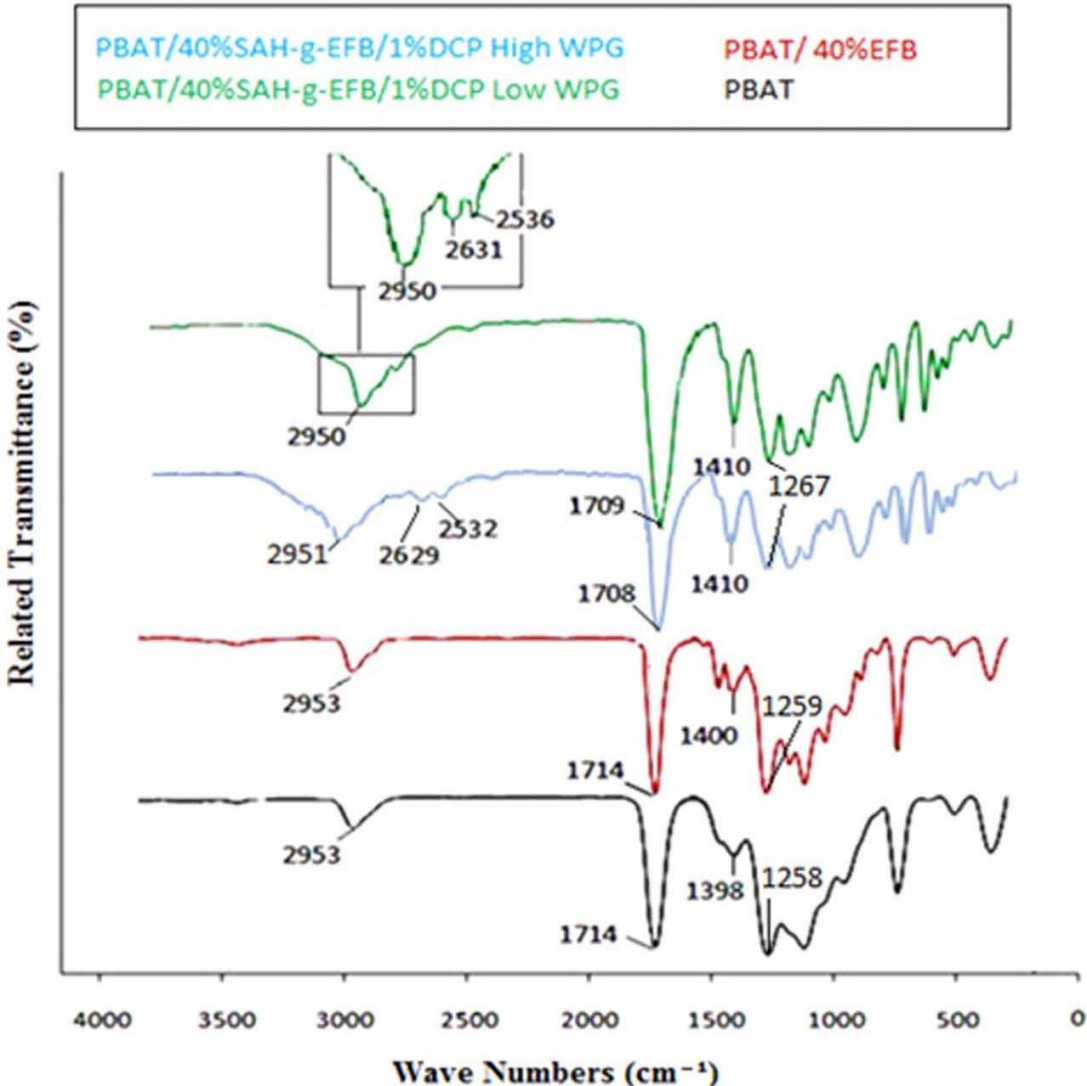
FT-IR spectra of the poly(butylene adipate-*co*-terephthalate) (PBAT) and its composites filled by untreated and esterified EFB fiber using succinic anhydride (SAH) and dicumyl peroxide (DCP) initiator.

Figure 2 depicts the FTIR spectra of the PBAT/EFB fiber composites before and after EFB fiber esterification using SAH and dicumyl peroxide (DCP) initiator, taken at low and high WPG. The changes in biocomposite as a result of the fiber esterification were followed by FT-IR spectroscopy. The spectra for both, modified and unmodified composites compared to the spectra of neat PBAT are shown in Figure 2. The PBAT composite formed by esterified fiber spectrum shows three new peaks between 2500 cm^−1^ and 3000 cm^−1^ regions. The peaks at about 2600 cm^−1^ could be due to vibrations of C–H (methyl groups) indicating the presence of alkyl chains and ester groups on the fiber produced by the anhydride modification of EFB fiber [13]. The SAH modified EFB fiber PBAT composites spectra clearly show broad intense peaks at 1708 cm^−1^ and 1709 cm^−1^ corresponding to the ester carbonyl groups (C=O) shifted from 1715 cm^−1^ in the PBAT spectra, evidencing successful esterification reaction in composites formed by pretreated fiber. In addition, the new band in EFB-g-SAH reinforced PBAT composites spectra at 1267 cm^−1^ corresponding to C=O stretching, further confirms the covalent linkage between SAH group and hydroxyl groups of the fibers [14]. Moreover, the FTIR spectrum of the treated biocomposite shows the dissimilarities between bands before and after fiber acetylation, wherever the major differences are related to finger print region from 1900 cm^−1^ to 600 cm^−1^ [15]. According to the FTIR spectra and with respect to the mechanical and physical properties of esterified EFB fiber reinforced PBAT polymer composite, we can conclude that DCP has likely initiated crosslinking reaction between EFB-g-SAH filler and PBAT matrix.

### 2.2. Effect of Fiber Loading on the Tensile Properties of Biocomposites

**Figure 3:**
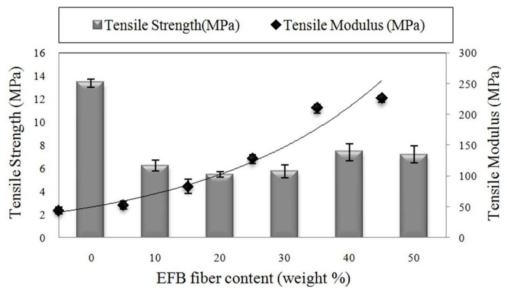
Effect of fiber content on tensile properties of PBAT biocomposites.

The PBAT/EFB composites with different fiber content were fabricated in order to determine the best formulation of the biocomposite. Figure 3 depicts the variation of the tensile strength as well as tensile modulus as a function of EFB fiber loading. Addition of EFB fiber decreased the tensile strength of PBAT, although there is a moderate increase in tensile strength of composite at 40% of fiber loading and, beyond this point, tensile strength decreased with the increase of fiber content. The highest tensile strength of 7.5 MPa was observed at 40% of fiber loading. The decline in tensile strength with increasing fiber content could be due to the lack of PBAT to wetten EFB fiber, resulting in an inefficient stress transfer [4]. This also might be due to the incompatibility of hydrophilic lignin in the EFB fiber with the matrix leading to an easy composite failure [16,17]. In composites with higher fiber content, there is a greater tendency for filler-to-filler interaction to take place [18]; therefore, more voids are formed, which cause the crack formation and propagation in the composite, compared with low fiber loading. Figure 2 also clearly illustrates that the biocomposites tensile modulus was considerably enhanced by fiber loading at high fiber content; the increased population of fiber leads to agglomeration, which affects the biocomposites’ stiffness. The overall increase in the modulus demonstrates the ability of the EFB fibers to impart greater stiffness to the composite [19]. Composite at 50 wt% of fiber content showed the highest tensile modulus of (225 MPa).

### 2.3. Effect of Fiber Chemical Treatment on Tensile Properties

To develop a biocomposite with acceptable mechanical performances, higher fiber loading composites need several modifications including chemical modification. Chemical modification of EFB fibers were successfully done by grafting SAH onto EFB fiber and the results were collected in two types of high and low WPG of EFB-g-SAH fibers. The WPG were increased as the reaction time was prolonged, and the WPG of esterified EFB fiber generated at 120 °C after 5 and 6 h were 17.75% and 31.28%, respectively. Table 1 shows the effects of WPG and various types and content of initiators on the tensile properties of 40 wt% of EFB-g-SAH fiber reinforced PBAT biocomposites.

A general increase in tensile strength of biocomposites was observed with the fiber esterification and increase of initiator content. The addition of esterified EFB fiber gradually increased the tensile strength of the biocomposite from 7.43 to 8.8 MPa for low WPG and to 8.4 MPa for high WPG SAH-g-EFB fiber reinforced PBAT biocomposites. Results show that the addition of organic peroxides as initiator whilst melt blending of components increased the tensile strength of biocomposite. The highest tensile strength of about 11 MPa was achieved in presence of 1 wt% of DCP initiator in composite reinforced with low WPG of esterified fiber. This could be due to the increase in the grafting rate or the formation of new bonds at the fiber and matrix interface at low concentration of DCP initiator. However, by further loading of DCP initiator beyond this concentration, tensile strength of the biocomposite is slightly decreased (in 1.5 wt% of DCP). The outcome might be due to the increase of homo-polymer formation in high organic peroxide concentration [20]. The effects of EFB fiber esterification in two levels of WPG by SAH in presence of various types and content of initiators on tensile modulus of new biocomposites are also presented in Table 1. It is clear that, using initiators to form new biocomposites with esterified EFB fiber at high WPG had no significant influence on the tensile modulus of PBAT biocomposites. Tensile modulus for biocomposite reinforced with low WPG of EFB-g-SAH fiber marginally increased from 210 to 220 MPa compared to that of untreated biocomposite. This is probably due to the modified fiber being more hydrophobic and hence exhibiting fiber matrix adhesion. Similar results are reported by Abdul Khalil and coworkers in their study on the effect of various anhydride modifications on mechanical properties of EFB fiber reinforced polyester composites [10]. The decrease in the tensile modulus of biocomposite after fiber esterification could be due to the reduction of fiber stiffness after introducing the SAH groups to the fiber surface, leading to the reduction of composites’ tensile modulus. It must be noted that the tensile modulus of composites decreased as the WPG increased.

A number of tests have been carried out regarding the effect of various anhydride modifications of rice husk fiber using SAH, maleic anhydride (MAH) and acetic anhydride (AAH) on the mechanical properties of RH-polyester composites. The lower performance demonstrated by the SAH modified composites, compared to that of MAH and AAH-treated composites, was due to the inability of the SAH to form crosslinking with the matrix [21]. Generally, in case of high WPG, further loading of succinic anhydride does not impart significant improvement in the tensile properties; in fact, the strength reduced as more SAH was introduced on the surface of EFB fiber. Thus, the introduction of SAH on the EFB fiber surfaces may produce a plasticizing effect, allowing movements at the fiber and matrix interface.

### 2.4. Effect of Fiber Loading on Flexural Properties of Untreated Composites

**Figure 4:**
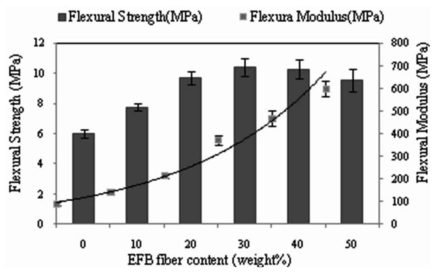
Effect of EFB fiber loading on the flexural properties of PBAT biocomposites.

Figure 4 depicts the effect of fiber loading on flexural strength and flexural modulus of EFB fiber reinforced PBAT biocomposites. As can be seen, both flexural strength and modulus show continuous increase with the increment of EFB fiber loading. This may be due to the ability of the EFB to absorb stress or the improvement in interaction between the fiber and matrix with the increase in fiber content. Figure 4 also shows that the percentage of EFB fiber loading has a similar effect on the flexural modulus as the tensile modulus. Since we use the modulus to measure the flexural stiffness of composite, using fillers improves the stiffness of composites [22]. As is seen in Table 2, there is a significant increase of about 75% and 417% in flexural strength and modulus, respectively, at 40% of EFB fiber loading.

Similar results were observed by Sykacek in their study on the mechanical performances of five biopolymer composites reinforced with wood flour. The best results were achieved by the wood flour reinforced Ecoflex^®^ composite. They declared that this difference is attributed to the applied multidirectional load, suggesting a greater impact of stiffness during flexural load [23]. Table 2 depicts the effect of fiber loading, fiber esterification and DCP initiator on the flexural properties of PBAT/EFB biocomposites with 40 wt% of succinylated fiber content. Even though the esterification of the fibers has little effect on the flexural strength of composite, a slightly higher (about 8%) flexural strength is seen with SAH-g-EFB fiber reinforced composite in presence of 1 wt% of DCP initiator. Thus, these fillers are not reinforcement; however they serve to separate regions of the polymeric matrix and a slight efficient stress transfer between the modified fiber and matrix [24].

In the case of flexural modulus, as can be seen in Table 2, esterification of EFB fiber with SAH has marginally increased the flexural modulus of biocomposite, indicating that the incorporation of modified fiber is partly able to instill stiffness into the PBAT matrix, and helps in better fiber dispersion. Fiber esterification, particularly in presence of DCP initiator, improved the flexural modulus of biocomposite by about 11% compared to the flexural modulus of unmodified fiber reinforced composite.

Therefore, modification of EFB fiber enhanced PBAT/EFB biocomposite compatibility and bonding characteristics through producing compatible surface energies as well as developing interface bonds. Similar trends were also reported by Hill and Abdul Khalil in their study on the effects of anhydride modification of fiber on the mechanical properties of Coir and EFB fiber biocomposites [24].

### 2.5. Effect of Fiber Loading and Chemical Modification on Thermal Properties of PBAT/EFB Biocomposites

**Figure 5:**
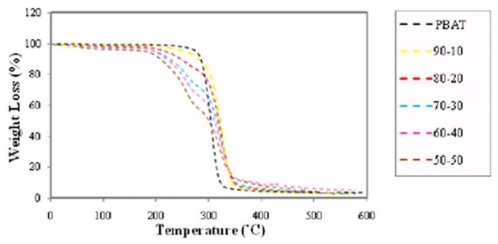
Effect of fiber content on thermal degradation of PBAT/EFB biocomposites (TG thermograms).

**Figure 6:**
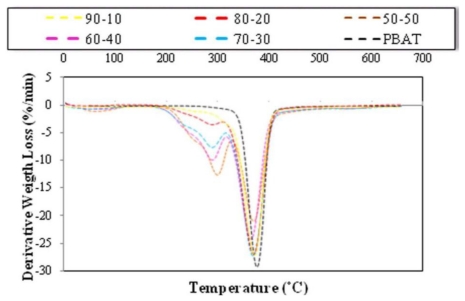
DTG thermograms of PBAT and PBAT/EFB composites in various fiber contents.

The TG and DTG thermograms of EFB fiber reinforced PBAT biocomposites in different fiber contents are presented in Figures 5 and 6, respectively.

Figures clearly show that the thermal stability of composites declined as the fiber content in composites improved. It is also obvious that the devolatilization of moisture of EFB fillers occurred in temperature range of 70–100 °C even after the EFB fillers incorporated into the PBAT matrix. Such moisture content might play a significant role in degradation processes due to the fact that OH group in water is more reactive than the OH group available in the EFB filler. The thermal degradation of EFB filler was due to the decomposition of cellulose, lignin and hemicellulose to give off volatiles [25]. Figure 6 demonstrates that PBAT starts to decompose at about 310 °C. Mass loss of PBAT happened in a one-step degradation procedure from 310 °C to less than 450 °C.

The mass loss of PBAT starts at about 310 °C and continues very slowly until 350 °C; at temperatures beyond 350 °C, this progression occurs quickly. The amount of PBAT residue is about 4.4% because of its further breakdown into gaseous products at high temperature. The only peak in DTG thermogram of PBAT at temperature of about 381 °C, is confirming the result (Table 3 and Figure 6).

Alternatively, the thermal degradation of the PBAT/EFB composites with various fiber contents takes place in a two-step degradation process (Figure 5), and the result is confirmed by the presence of two peaks from DTG thermogram (Figure 6). Besides, mainly due to the decomposition of EFB fiber, the PBAT/EFB biocomposites exhibited initial mass loss from approximately 207 °C to 340 °C. Then, the second thermal degradation step, which is mainly related to PBAT degradation, overlapped with cellulose and lignin content in EFB fiber is observed. This two-step degradation process demonstrates that the thermal degradation temperature of the PBAT is higher than EFB fiber’s. Furthermore, Table 4 clearly shows that, the formation of EFB biocomposites using hydrophobic PBAT matrix reduced water content of biocomposites. PBAT surrounds EFB fiber and its presence reduces fiber hydrophilicity and decreases the water content in biocomposite via preventing water to reach the EFB fiber.

From the data presented in Table 4 we can conclude that the intermediate values of degradation temperature continue to suggest some kind of interaction between the PBAT matrix and fiber. Based on the wt% of EFB content, the thermal stability of PBAT/EFB fiber composites follows the sequence of 10% > 20% > 30% > 40% > 50% [15].

### 2.6. Effect of Chemical Modification on Thermal Properties of PBAT/EFB Fiber Composites

**Figure 7:**
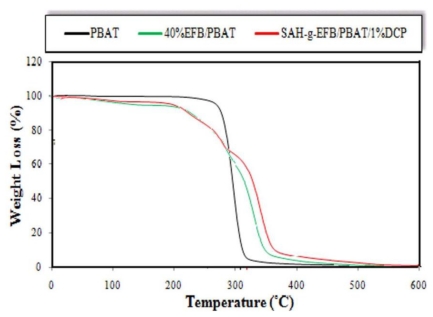
Effect of SAH modification on thermal degradation rate of PBAT/EFB biocomposites (TG thermograms).

The (TG) thermograms and the TG details of experimental data for PBAT and its composites filled by 40 wt% of EFB fiber, before and after chemical modification with SAH in presence and absence of DCP initiator, are shown in Figure 7 and Table 5, respectively.

From the TGA data, it is clear that the water content of PBAT/EFB biocomposite decreased after chemical treatments on fiber compared to that of the composite without chemical additive and coupling agent. As stated earlier, forming EFB biocomposites using hydrophobic (PBAT) matrix reduces the composites water content. Moreover, chemical treatments have also reduced the biocomposites water content. Chemical treatment turns fiber surface hydrophobic, and fibers functional groups surrounded with hydrophobic PBAT inhibit water penetration into the composite structure.

In order to verify the interactions between the degradation mechanisms affecting the ash residues, the percentage of weight loss at 550 °C was measured (Table 5). The char formation, mostly correlated to the fiber (producing 29% and 3.8% of char residues for pure EFB fiber and PBAT, respectively), follows this order for the materials studied in this work: PBAT/SAH-g-EFB/DCP > PBAT/40%EFB composite. Chemically treated biocomposites presented more ash residues compared to the composite without coupling agent, possibly because of the increased interaction between the polymer matrix and fiber through the degradation procedure [8].

**Figure 8:**
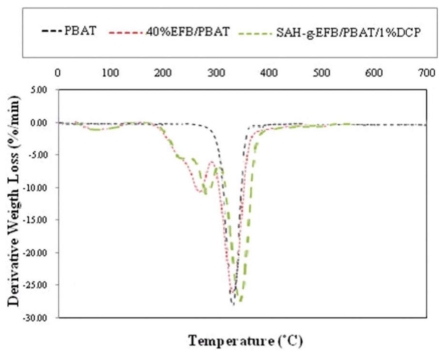
DTG thermograms of PBAT and PBAT/EFB composites before and after SAH/DCP modification.

Figure 8, shows three stages of thermal degradation for esterified EFB fiber biocomposite. The esterification of EFB fibers increased the thermal stability of biocomposite compared to the untreated EFB fiber reinforced composite. The esterified EFB fiber composite had an initial weight loss temperature ranging from 180 °C to 250 °C with 8.5% weight loss, which is lower than that of untreated composite. The second weight loss (17%) occurred at about 316.6 °C (Table 6), and could be due to the onset of pyrolysis of lignin content in the composite’s filler.

The third weight loss of about 52.5%, observed at about 388.2 °C, is very comparable to that of the untreated composite and may be due to the decomposition of cellulose overlapped PBAT matrix. The initial weight loss may be due to de-carboxylation of the introduced carboxyl groups by grafting SAH onto EFB fiber overlapped fiber hemicellulose content. Increasing the monoester or ester content may result in a decline in the initial weight loss temperature and the greatest mass loss temperature [26]. These outcomes point out a de-esterification reaction of the carboxyl groups or a de-carboxylation reaction followed by de-carboxylation, or both.

From Tables 5 and 6, it is obvious that the thermal degradation of untreated biocomposite starts at about 153.3 °C, and finishes at 443.6 °C with a main peak at 380.9 °C. The mass loss before the onset temperature was correlated to the volatilization of fiber water content. Therefore, at the onset temperature of the neat PBAT and composites, with and without SAH coupling agent, the difference of mass loss was mostly because of the EFB contents and SAH, which had a significant effect on mass loss at onset temperature of treated biocomposite. The decomposition temperature of PBAT was around 380 °C, its complete decomposition has taken place at about 400 °C. However, the decomposition temperature of PBAT rose to about 380.9 °C and 388.2 °C with the addition of unmodified and modified fiber, respectively.

### 2.7. Morphological Study of PBAT/EFB Biocomposite at 40% Fiber Loading

**Figure 9:**
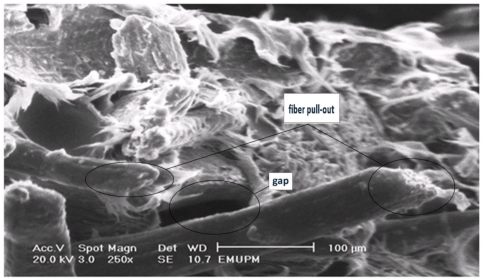
The tensile fracture surface micrograph of PBAT biocomposite reinforced with 40 wt% of EFB.

Figure 9 illustrates the scanning electron micrograph of tensile fracture surface of PBAT/EFB composites at 40 wt% of fiber content. At first sight, the dispersion of fibers in the matrix, regardless of the employed surface treatment is clear, proving the efficient mixing of fibers in the matrix via melt blending of fibers and PBAT in Rheomixer. As seen in Figure 9, the EFB fiber composite fracture occurred, predominantly, by transversal fracture in the flat surface of the PBAT matrix. It is also observed that the EFB particles in fiber form oriented in random arrangements, and as the adhesion fiber/matrix was poor, the composite shows some gaps and signs of a pullout tendency. In addition to fiber pullout, some fiber breakage as the mean of fracture can also be seen in the PBAT/EFB untreated sample.

### 2.8. Morphological Study of PBAT/EFB Biocomposite after Fiber Modification

**Figure 10:**
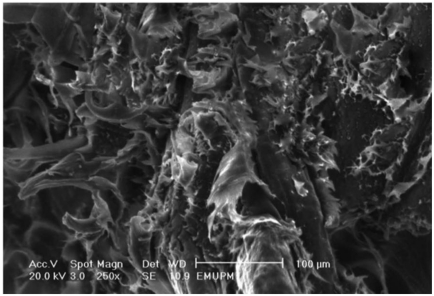
Tensile fracture surface micrographs of low WPG esterified EFB fiber reinforced PBAT biocomposite in presence of 1 wt% of DCP initiator.

Figure 10 illustrates the scanning electron micrograph of tensile fracture surface of PBAT composites reinforced with (Low WPG) esterified EFB fiber at 40 wt% of fiber content in presence of DCP initiator. Figure 10 demonstrates that the esterification of EFB fiber surfaces has changed the fracture mode of PBAT/SAH-g-EFB composite and reduced voids around the fibers. Some fibers were coated with the matrix after treatments although there was fiber pullout. Comparison of esterified EFB fiber composite surface with that of the composite before treatment shows that surface roughness of composites after treatment is lower than that of untreated composites. Decreased fracture surface roughness of composite with fiber esterification is a direct evidence of decreased path distance during crack propagation and decreased toughness of composites, accordingly [27].

### 2.9. Effect of Fiber Loading and Fiber Chemical Modification on Water Absorption of PBAT/EFB Biocomposites

**Figure 11:**
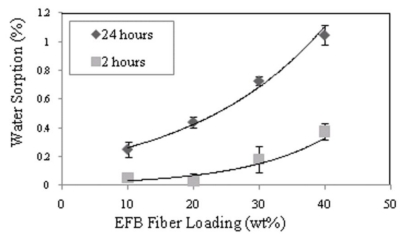
Effect of EFB fiber loading on water sorption of PBAT/EFB composites.

Figure 11 illustrates the effects of fiber loading on water absorption of PBAT/EFB biocomposites. As expected, addition of EFB fiber to the neat PBAT polymer to form biocomposites increased the water absorption of the biocomposites. Weight gains in the range of 0.05% to 0.38% and 0.25% to 1.05% were obtained after 2 and 24 h immersion in distilled water, respectively. The highest water uptake rate belonged to PBAT biocomposite reinforced with 40 wt% of untreated EFB fiber, which was mostly because of the hydrophilic nature of the EFB fibers due to the existence of polar groups like hydroxyl, acetal and ether linkages in the fibers’ cellulose structure. To form a hydrogen bond, the free hydroxyl groups in cellulose and lignin structure interact with the water molecules. Porous structure of the EFB fiber surface facilitated the water penetration into the fiber by capillary action. This also might be attributed to the swelling of the fibers when it is exposed to water which leads to crack formation. Micro-cracks can produce pathways for easier diffusion of the water molecules into the composite and thus improve their water absorption mechanism [28].

**Figure 12:**
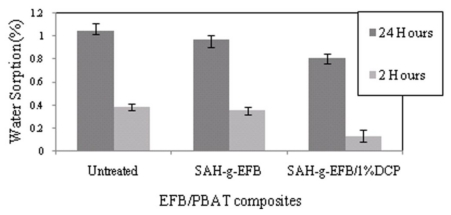
Effects of chemical treatments on water absorption of PBAT/EFB biocomposite.

Effects of chemical treatment using SAH in presence and absence of DCP initiator on water absorption of PBAT/EFB fiber biocomposite after 2 and 24 h immersions in distilled water is presented in Figure 12. The percentage of EFB fiber by mass in the biocomposites was 40%. As seen in Figure 12, EFB fiber treated with SAH reduced the water absorption of PBAT/40%EFB-g-SAH/1%DCP biocomposite (about 24%) compared to that of the untreated composite. The decrease in water uptake of the SAH treated composite is due to the esterification reaction, which caused the reduction of hydroxyl group on the fiber surface [7], and therefore, fibers became more hydrophobic. Modification of the fiber cell-wall hydroxyl groups reduced moisture uptake and this is attributed to enhanced matrix-fiber contact [10].

## 3. Experimental

### 3.1. Materials

EFB fiber was purchased from Sabutek Sdn Bhd, Malaysia, in form of short fiber with an average length of 5 mm. The EFB fibers, consisting of 65% cellulose and 19% lignin, were obtained after the removal of oil seeds from fresh fruit bunch used for oil extraction [20]. The fibers were ground and then sieved to the size of 100–200 μm (size of a single fiber in length). The sieved fibers were soaked for 3 h and then washed with warm distilled water and acetone, in order to remove impurities, dust and oil from the fibers surface. The washed fibers were then dried in an air oven at 50 °C until reaching the constant weight.

Aliphatic aromatic *co*-polyester (PBAT), with the trade name of Ecoflex^®^ FBX 7011, was purchased from BASF Plastic Technologies USA. The succinic anhydride (SAH), in form of white crystalline solid was supplied by ACRŌS (ORGANICS) Geel (Belgium). Reagent grade chemicals, namely dicumyl peroxide (DCP) and benzoyl peroxide (BPO), used for chemical modification as initiators in producing composites, were provided by Aldrich Chemical Co. Ltd. The chemicals of analytical grades were used through the research.

### 3.2. Fiber Chemical Treatment

Approximately 16 g of sieved oven dried EFB fibers were placed in a 500 mL round bottom reaction flask containing 150 mL of the preheated succinic anhydride: pyridine solution and fitted to a reflux condenser. The reaction flask was heated at 120 °C for either 5 or 6 h in the oil bath. Upon reaching allocated time, 300 mL of acetone was added and refluxed for another 1 h at 56 °C to quench the reaction. Finally, to remove solvent and unreacted reagents, another 150 mL of acetone was added into the reaction flask and left at ambient temperature until the reaction subsided. The treated fibers were then oven dried at 103 °C overnight, cooled in a desiccator over silica gel and weighted. The percentage of weight gain (based on percentage of oven-dried weight) of chemically modified fibers was then calculated as (1) follow. Proof of whether modification had taken place depended on the WPG value [10]:

(1) $$\text{WPG }(\%)=\frac{\text{weight gain}}{\text{original weight}}\times 100$$

### 3.3. Preparation of the Composites

To study the effect of fiber loading on the thermal and mechanical properties as well as the water absorption of PBAT biocomposites, the compounding was carried out at different fiber loading of 10, 20, 30, 40 and 50 wt%. PBAT biocomposites reinforced with 40 wt% of EFB-g-SAH fiber were developed in presence and absence of dicumyl peroxide (DCP) and benzoyl peroxide (BPO) as organic initiators, to study the changes in the mechanical and thermal properties as well as the water absorption of biocomposites. The melt blending procedure carried out at 120 °C by Thermo Haake PolyDrive internal mixer at 30 rpm of speed. The composite samples were preheated at 135 °C without applying any pressure for 35 min to allow complete melting. The melted compound was then pressed to mould into sheet at the same temperature.

### 3.4. FTIR Spectroscopy

A FTIR spectrophotometer was used to determine the functional group in the samples. FTIR measurements can confirm chemical structure of biocomposites. FTIR spectra tests were run at ambient temperature using a KBr disk method at wave number range of 400 to 4000 cm^−1^, resolution of 4 cm^−1^. The infrared spectra of samples were measured on a Perkin-Elmer FTIR (model spectrum 100 series).

### 3.5. Biocomposites characterization

#### 3.5.1. Mechanical Testing of Biocomposites

The produced biocomposite sheets were cut into four standard types of samples for tensile and flexural tests. Tensile tests were carried out according to ASTM Standard Method D638-99 [29] on dumbbell shape specimens with 1mm thickness, using an Instron Universal Tester (model 4302) at 5 mm/min crosshead speed. The flexural test was performed using the same machine according to ASTM D790-07 [30] on rectangular standard specimens with the dimension of 120 mm × 12.7 mm and 3 mm thickness. Tests were performed at room temperature (about 25 °C) and a minimum of seven samples were tested in each case.

#### 3.5.2. Thermal Behavior (Thermogravimetric Analysis, TGA)

Neat PBAT and its biocomposites, before and after chemical treatment, were subjected to thermogravimetric analysis using Perkin-Elmer Thermal Analyzer (model TGA 7). The tests were carried out at heating rate of 10 °C/min under nitrogen atmosphere with flow rate of 20 mL/min. The onset temperature of a 3% (T_3_%) weight loss deviation from the baseline of the thermogravimetric (TG) thermogram was used as the indicator of the composite’s thermal stability and the differential thermogravimetric (DTG) thermograms were recorded to study the weight loss.

#### 3.5.3. Morphological Features

Tensile fracture surfaces of the composites were examined to evaluate the fiber/matrix adhesion and survey the effects of chemical treatment in different biocomposites, using a scanning electron microscope model LEO 1455VP SEM analyzer. All the surfaces were examined after they were gold coated using a Bal-Tec SDC005 coater sputter.

#### 3.5.4. Water Absorption Test

The water absorption test followed ASTM standard test method D570-98 [31]. Prior to measurement, the specimens were dried in an air oven at 50 °C for 24 h, cooled in a desiccator over silica gel and then weighed immediately with a precision of 1 mg, the weight was then taken as the dry initial weight of the sample (*M*_0_). The specimens were then immersed in a container of distilled water for 24 h. Water uptake of PBAT/EFB fiber composites at time *t* was calculated as follow:

(2) $$\text{Uptake }\%=(M_{\text{t}}-M_{0})/M_{0}\times 100$$

where, *M*_t_ = Mass of sample at time *t*.

## 4. Conclusions

Biodegradable composites based on 40 wt% esterified EFB fiber have been successfully prepared via melt blending technique and characterized. The succinylated fiber yields improved tensile and flexural properties of the composite in presence of 1% DCP initiator. The mechanical findings corroborated morphological evidence. The fiber chemical treatment using SAH in presence of 1% DCP at 40% fiber loading also improved the fiber-matrix interaction, which led to better incorporation of fiber with the matrix. TGA characterization shows improved thermal stability for PBAT/EFB-g-SAH biocomposite in presence of DCP initiator, compared to that of PBAT/EFB biocomposite. SEM micrographs demonstrate a better dispersion of EFB fiber into the matrix. SEM also provides evidence that the esterification of EFB fiber surfaces has reduced voids around the fibers, increased interfacial adhesion and presents a smoother surface.

Compared to that of the unmodified composite, EFB fiber treated with SAH reduced the number of hydroxyl groups on the EFB fiber surface and therefore decreased water absorption of PBAT/40% EFB-g-SAH/1%DCP biocomposite up to about 24%. In fact, the esterification of the fiber cell-wall hydroxyl groups led to better fiber-matrix contact and enhanced biocomposites’ water resistance.

## Figures and Tables

**Table 1:** The effects of WPG and various type and content of initiators on the tensile properties of EFB-g-SAH fiber reinforced PBAT biocomposites.

	PBAT/40 wt% EFB-g-SAH (High WPG *)				PBAT/40 wt% EFB-g-SAH(Low WPG *)			
	
	Tensile Strength (MPa)		Tensile Modulus (MPa)		Tensile Strength (MPa)		Tensile Modulus (MPa)	
**Peroxide Content (%)**	BPO ***	DCP	BPO	DCP	BPO	DCP	BPO	DCP
0	8.4 (0.77)**	8.4 (0.77)	109 (9.11)	109 (9.18)	8.8 (0.31)	8.8 (0.31)	154 (8.11)	154 (8.10)
0.5	9.0 (0.53)	8.8 (0.73)	139 (7.83)	156 (6.53)	9.9 (0.26)	10.2 (0.36)	194 (8.95)	197 (9.42)
1	9.6 (0.44)	9.6 (0.91)	143 (8.30)	176 (8.94)	10.1 (0.44)	11.0 (0.29)	200 (9.05)	220 (9.10)
1.5	9.8 (0.60)	9.2 (0.61)	134 (8.25)	176 (7.20)	10.2 (0.63)	10.4 (0.62)	198 (7.78)	202 (7.51)

* WPG is weight percent gain.

** The standard deviations are given in parentheses.

*** The (wt%) of benzoyl peroxide (BPO) and DCP contents calculated based on fiber content weight in each composite.

**Table 2:** Effects of fiber loading, fiber esterification and DCP initiator on the flexural properties of PBAT biocomposites.

Samples	Flexural Strength (MPa)		Flexural Modulus (MPa)	
**PBAT**	5.9	(0.31) *	90	(4.28)
**PBAT/EFB 10 wt% of fiber**	7.8	(0.26)	140	(5.12)
**PBAT/EFB 20 wt% of fiber**	9.7	(0.44)	214	(9.61)
**PBAT/EFB 30 wt% of fiber**	10.0	(0.58)	371	(2.32)
**PBAT/EFB 40 wt% of fiber**	10.3	(0.63)	465	(3.51)
**PBAT/EFB 50 wt% of fiber**	9.5	(0.76)	596	(3.50)
PBAT/40 wt% EFB-g-SAH**	10.4	(0.92)	473	(2.91)
PBAT/40 wt%EFB-g-SAH/DCP***	11.1	(1.04)	518	(2.21)

* The standard deviations are given in parentheses.

** The esterified fiber used at low weight percentage gain.

*** The 1 wt% of DCP content calculated based on fiber content weight in each composite.

**Table 3:** Summary of DTG_max_ degradation temperature of PBAT and PBAT/EFB biocomposites with various fiber contents.

Specimens	First peak (°C)	Second peak (°C)	Third peak (°C)
PBAT	–	–	379.6
PBAT/EFB10%	–	–	386.1
PBAT/EFB20%	–	305.5	383.3
PBAT/EFB30%	88.0	307.5	382.2
PBAT/EFB40%	88.8	307.0	380.9
PBAT/EFB50%	79.5	307.2	379.4

**Table 4:** Summary of thermogravimetric analysis (TGA) data for PBAT and PBAT/EFB biocomposites with various fiber contents.

Sample	Water content (%)	Initial degradation temperature (°C) *	T_10%_	T_50%_	T_80%_	Final degradation temperature (°C)	Ash content (%) *
PBAT	–	311.5	352.8	381.3	398.4	413.3	4.4
PBAT/EFB10%	–	280.4	339.7	382.8	399.2	431.7	6.7
PBAT/EFB20%	1.3	248.8	302.3	378.0	398.6	432.5	9.1
PBAT/EFB30%	2	210.2	276.7	373.6	401.5	440.2	11.2
PBAT/EFB40%	3.9	153.3	275.2	371.1	400.2	443.6	12.2
PBAT/EFB50%	3.4	148.2	233.3	360.2	398.8	447.7	17.7

Note:

* The initial degradation temperature considered as the temperature when the sample loses 3% of its weight.

** The ash content is the weight percentage at 550 °C TGA data for PBAT/EFB biocomposites.

**Table 5:** Summary of TGA data for PBAT/EFB biocomposites with various chemical modifications using SAH/DCP.

Sample	Water content (%)	Initial degradation temperature (°C) *	T_10%_	T_50%_	T_80%_	Final degradation temperature (°C)	Ash content (%) **
Neat PBAT	–	311.5	352.8	381.3	398.4	413.3	4.4
PBAT/40%EFB	3.9	153.3	275.2	371.1	400.2	443.6	12.2
PBAT/EFB-g-SAH/1%DCP	2.0	136.8	267.1	378.7	446.4	450.2	14.8

Note:

* The initial degradation temperature considered as the temperature when the sample loses 3% of its weight.

** The ash content is the weight percentage at 550 °C.

**Table 6:** Summary of DTG_max_ degradation temperature of PBAT/EFB biocomposites.

Specimens	First peak (°C)	Second peak (°C)	Third peak (°C)	Fourth peak (°C)
PBAT	–	–	–	379.6
PBAT/40%EFB	88.8	–	307.0	380.9
PBAT/40%EFB-g-SAH/1%DCP	86.5	249.7	316.6	388.2
